# Breast Cancer Surgery: Past, Present and Future—A Narrative Review

**DOI:** 10.3390/jcm15103778

**Published:** 2026-05-14

**Authors:** Paolo Izzo, Marcello Molle, Pierfrancesco Di Cello, Paolo Meloni, Silvia Lai, Luciano Izzo, Simone Sibio, Daniela Messineo, Sara Izzo

**Affiliations:** 1 “Pietro Valdoni” Department of Surgery, Policlinico “Umberto I”, Sapienza University of Rome, 00161 Rome, Italy; luciano.izzo@uniroma1.it (L.I.); simone.sibio@uniroma1.it (S.S.); 2 Plastic and Reconstructive Surgery Unit, Multidisciplinary Department of Medical-Surgical and Dental Specialties, Università degli Studi della Campania “L.Vanvitelli”, 80138 Naples, Italy; marcello.molle@unicampania.it (M.M.); sa_izzo@hotmail.it (S.I.); 3 General Surgery Unit, ASL Frosinone, 03100 Frosinone, Italy; pf4@libero.it; 4 Department of Radiological, Oncological, and Anatomical Pathology Sciences, Sapienza University of Rome, 00161 Rome, Italy; paolo.meloni@libero.it; 5 Nephrology Unit, Department of Translational and Precision Medicine, Policlinico “Umberto I”, Sapienza University of Rome, 00161 Rome, Italy; silvia.lai@uniroma1.it; 6 Department of Radiological Sciences, Oncology and Anatomo-Pathological Science, Sapienza University of Rome, 00161 Rome, Italy; daniela.messineo@uniroma1.it

**Keywords:** breast cancer surgery, mastectomy, sentinel lymph node biopsy, neoadjuvant systemic therapy, oncoplastic surgery, robotic mastectomy, intraoperative radiotherapy, artificial intelligence

## Abstract

Breast cancer surgery has evolved from radical procedures to increasingly individualized and less invasive approaches. This narrative review contextualizes this evolution, synthesizes current evidence supporting surgical de-escalation, and examines emerging strategies that may further reduce the need for surgery. The manuscript is based on a structured appraisal of PubMed/MEDLINE literature and major international guidelines, prioritizing randomized trials, prospective studies, and consensus statements. Contemporary practice is characterized by progressive reduction in both breast and axillary surgery, enabled by advances in tumour biology, neoadjuvant systemic therapy, sentinel node strategies, and oncoplastic techniques. Emerging approaches—including selective omission of axillary surgery, targeted axillary dissection, and investigational strategies aiming at omission of breast surgery in exceptional responders—highlight a shift toward response-adapted and biology-driven care. While technological innovations such as robotic surgery and intraoperative radiotherapy may influence surgical practice, their role in true de-escalation remains limited or context-dependent. Overall, the field is moving toward minimizing surgical burden without compromising oncological safety, with future progress likely driven by improved patient selection, imaging, and integration of systemic therapy response.

## 1. Introduction

Breast cancer remains the most diagnosed malignancy among women worldwide. In the United States, a large proportion of patients present with localised-stage disease, for which treatment has become progressively less extensive while maintaining excellent outcomes [1]. Indeed, the 5-year relative survival rate for localised breast cancer now exceeds 99%, reflecting major advances in screening, systemic therapies, and surgical approaches [1]. The evolution of breast cancer surgery is largely characterised by a progressive de-escalation of treatment. This shift began with the en bloc radical mastectomy introduced by William Stewart Halsted in the late nineteenth century and continued through the development of modified radical mastectomy in the mid-twentieth century. By the late twentieth century, breast-conserving surgery (BCS) had been established as oncologically equivalent to mastectomy for appropriately selected patients with early-stage disease. This trend toward less invasive management persists today, with strategies such as omission of completion axillary dissection in selected cases, increasing use of oncoplastic techniques, and ongoing investigation into the potential omission of surgery in exceptional responders to neoadjuvant systemic therapy (NST) [2,3,4].

This narrative review provides an overview of the historical evolution, current practices, and future directions of breast cancer surgery, with a particular focus on practice-changing clinical trials, contemporary de-escalation strategies, and emerging technologies.

## 2. Materials and Methods

This study was conducted as a structured narrative review. The literature informing the manuscript was identified through a focused appraisal of PubMed/MEDLINE and major international guideline documents relevant to breast-conserving surgery, mastectomy, axillary management, neoadjuvant systemic therapy, radiotherapy, oncoplastic surgery, robotic surgery, and emerging digital technologies.

The search strategy combined controlled vocabulary and free-text terms related to breast cancer surgery, with particular emphasis on de-escalation strategies and surgical innovation. Landmark historical publications were included irrespective of publication date when necessary to contextualise major paradigm shifts in surgical management.

Priority was given to landmark randomised controlled trials, prospective multicentre studies, consensus guidelines, and high-quality review articles considered either practice-defining or hypothesis-generating. Studies were selected based on their relevance to the evolution of surgical de-escalation, contemporary clinical decision-making, and future directions in breast oncology.

Given the interpretative aim of this review, rather than exhaustive study identification, a formal systematic review methodology—including pooled quantitative analysis and risk-of-bias assessment—was not applied. The narrative synthesis was developed with attention to transparency, balance, and clinical applicability, in line with established standards for high-quality narrative reviews [5].

**Figure 1:**
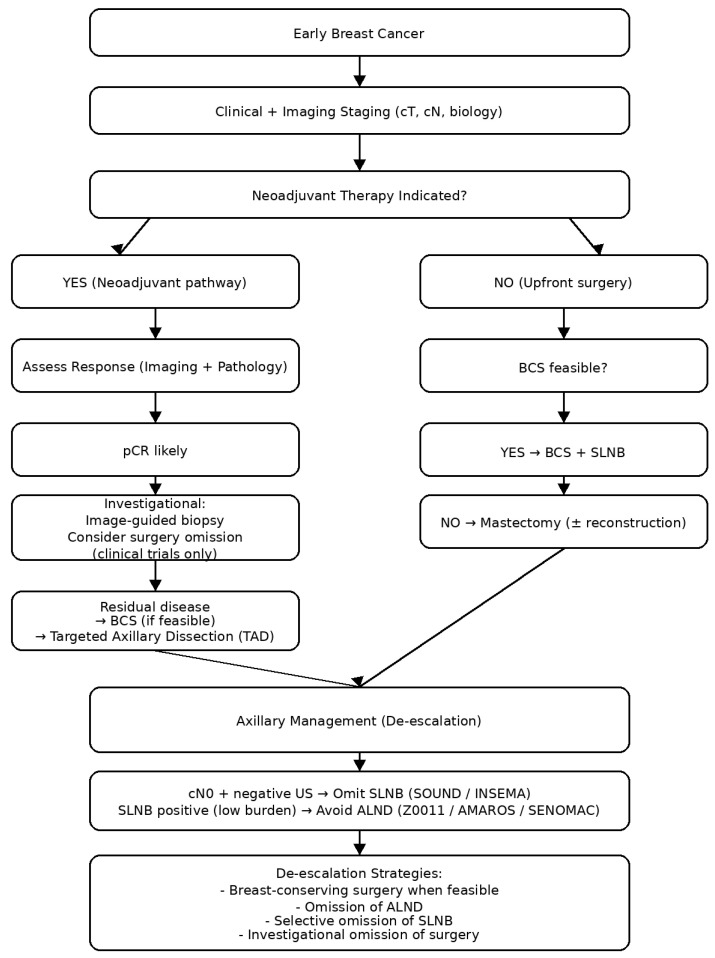
Evidence-based pathways for surgical de-escalation in early breast cancer. The diagram illustrates how tumour biology, response to neoadjuvant therapy, and nodal status guide the progressive reduction in breast and axillary surgery. Established strategies include breast-conserving surgery, omission of axillary lymph node dissection, and selective omission of sentinel lymph node biopsy, while omission of breast surgery remains investigational. (BCS = breast-conserving surgery; SLNB = Sentinel lymph node biopsy; ALND = axillary lymph node dissection); pCR = pathological complete response).

- Results (Figure 1)

## 3. Historical Perspective: From Radical to Conservative Surgery

### 3.1. The Halstedian Era

Modern surgical treatment of breast cancer began with William Stewart Halsted, who in 1894 described the radical mastectomy: en bloc removal of the breast, the pectoralis major and minor muscles, and the ipsilateral axillary lymph nodes [6]. The procedure dominated practice for decades and was based on the anatomical hypothesis that breast cancer spread in an orderly centrifugal fashion through contiguous lymphatic pathways. Although radical mastectomy improved locoregional control, it did so at considerable cost in terms of deformity, shoulder dysfunction and chronic lymphoedema.

### 3.2. The Paradigm Shift: Fisher and Veronesi

The biological model advanced by Bernard Fisher in the 1970s fundamentally challenged the Halstedian paradigm. Fisher proposed that breast cancer is frequently a systemic disease at presentation and that differences in locoregional treatment would not necessarily alter the risk of distant metastasis. This hypothesis was supported by two landmark randomised trials—NSABP B-06 and Milan I—both of which demonstrated equivalent long-term survival for breast-conserving treatment and mastectomy in selected patients with early-stage disease [7,8].

### 3.3. The Evolution of Axillary Surgery

Axillary management has followed a similar path of de-escalation. Sentinel lymph node biopsy (SLNB), first developed and validated in the early 1990s, progressively replaced routine axillary lymph node dissection (ALND) in clinically node-negative patients, with substantial reductions in lymphoedema and shoulder morbidity [9,10]. Subsequent trials, including ACOSOG Z0011, AMAROS and IBCSG 23-01, showed that completion ALND could often be omitted in patients with limited sentinel-node disease without compromising oncological safety [11,12,13].

## 4. Current Surgical Practice: Evidence-Informed De-Escalation

### 4.1. Trends in Breast Surgery (2010–2023)

Large contemporary registry analyses indicate an ongoing shift towards less extensive breast and axillary surgery [3]. Across the last decade, mastectomy and ALND rates have decreased, whereas the use of immediate reconstruction has increased. At the same time, omission of axillary surgery in carefully selected low-risk patients has become more common, reflecting growing confidence in de-escalated strategies and alignment with initiatives aimed at reducing low-value surgical care [3,4].

### 4.2. Breast-Conserving Surgery and Oncoplastic Techniques

BCS remains a reasonable surgical approach for most patients with early-stage breast cancer when negative margins and acceptable cosmesis can be achieved. For invasive carcinoma, the accepted margin standard is ‘no ink on tumour’ [14]. For ductal carcinoma in situ-treated with whole-breast irradiation, a 2 mm margin remains the most widely accepted benchmark [15]. Local recurrence after BCS followed by radiotherapy is low in the modern era, although risk varies according to tumour biology, adjuvant therapy and duration of follow-up [2].

The integration of oncoplastic techniques—combining oncological resection with plastic surgical principles—has expanded the indications for BCS to include larger tumours and anatomically challenging lesions. Volume displacement procedures, such as therapeutic mammoplasty, and volume replacement procedures, including local or regional flaps, can permit wider excision while maintaining or even improving breast shape. These approaches may reduce re-excision and completion mastectomy rates in selected patients [16,17]. Successful BCS increasingly depends on accurate localization of non-palpable lesions. Wire-guided localization has historically been the standard approach, but several alternatives are now available, including magnetic seed localization, radar reflector localization, radiofrequency identification tags, radioactive seed localization, and intraoperative ultrasound. These techniques may improve scheduling flexibility, patient comfort, and multidisciplinary workflow while maintaining adequate surgical accuracy. Their use remains institution-dependent and is influenced by local expertise, regulatory issues, availability, and cost [18,19].

### 4.3. Axillary Surgery in 2024–2025: Beyond SLNB

Axillary surgery is becoming increasingly individualised, moving away from a uniform radical approach [20]. The SOUND trial demonstrated non-inferiority of omitting surgical axillary staging, as compared with SLNB, in selected patients with clinically node-negative disease and negative axillary ultrasound findings, in a population composed predominantly of postmenopausal women with oestrogen receptor-positive/HER2-negative tumours [20,21]. The primary results of INSEMA and the 2025 ASCO guideline update have further reinforced the principle that surgical axillary staging can be omitted in carefully selected patients undergoing breast-conserving treatment [22,23].

For patients with clinically node-negative disease and limited sentinel-node metastases, completion ALND can often be avoided; however, the applicability of this approach depends on the operative context, the use of regional nodal irradiation and the burden of nodal disease. Recent evidence from SENOMAC has extended support for omission of ALND in selected patients with one or two macrometastatic sentinel nodes [24]. In the post-neoadjuvant setting, targeted axillary dissection (TAD)—combining retrieval of a previously clipped node with SLNB—has emerged as an important strategy for reducing surgical morbidity while maintaining staging accuracy. Prospective studies have shown that retrieval of the clipped node and use of dual tracers improve false-negative rates after NST [25,26,27], and ongoing trials such as TAXIS continue to explore how far axillary surgery can be safely de-escalated [20].

### 4.4. Neoadjuvant Systemic Therapy and Its Surgical Implications

NST is now the preferred initial treatment sequence for many patients with triple-negative breast cancer (TNBC) and HER2-positive disease. Effective neoadjuvant treatment can downstage the breast sufficiently to convert some patients who would otherwise require mastectomy into candidates for BCS [2]. With contemporary regimens, pathological complete response (pCR) rates are particularly high in HER2-positive disease and TNBC, and these biological responses increasingly influence surgical planning [2,28,29].

The addition of immunotherapy to neoadjuvant chemotherapy has further improved pCR rates in TNBC. The KEYNOTE-522 trial demonstrated that pembrolizumab plus chemotherapy increased pCR and improved event-free survival as compared with chemotherapy alone in early-stage TNBC [30,31]. More recently, phase III studies such as CheckMate 7FL and KEYNOTE-756 have reported higher pCR rates in selected patients with high-risk oestrogen receptor-positive/HER2-negative disease, although the long-term impact on survival and surgical de-escalation remains to be fully defined [32,33].

pCR is strongly associated with improved long-term outcomes, particularly in TNBC and HER2-positive disease [29]. These data support response-adapted escalation or de-escalation strategies in the postoperative setting, while also reinforcing the principle that surgical planning should be integrated with tumour subtype, imaging response and pathological assessment.

## 5. Emerging Frontiers and Future Directions

### 5.1. Robotic Nipple-Sparing Mastectomy

Robotic nipple-sparing mastectomy (RNSM), first described by Toesca and colleagues, represents a notable technological development in breast surgery [34]. By using a robotic platform through a small axillary or inframammary incision, the procedure seeks to minimise visible breast scarring while preserving the oncological principles of nipple-sparing mastectomy. Early comparative studies suggest similar short-term complication profiles to conventional approaches, albeit with longer operating times, higher costs and a substantial learning curve [35].

A recent meta-analysis suggested that RNSM may be associated with lower rates of selected wound-related complications and favourable patient-reported outcomes, although the available evidence remains largely non-randomised and heterogeneous [36]. Early single-port series and pooled multicentre analyses have also reported encouraging short-term and sensory outcomes when the dissection is performed through an axillary approach, but claims of complete sensory preservation should be avoided until validated in larger studies [37,38,39,40,41]. Contemporary consensus statements support the feasibility of RNSM in experienced centres, while emphasising the need for long-term oncological follow-up and robust prospective data [42].

### 5.2. Omission of Surgery in Exceptional Responders

One of the most provocative frontiers in breast cancer surgery is the possible omission of surgery in patients who achieve an exceptional response to NST. With modern treatment regimens, pCR rates can be high in TNBC and HER2-positive disease [43]. The key challenge is the accurate identification of pCR without definitive surgical excision. Image-guided vacuum-assisted biopsy of the tumour bed has therefore been investigated as a potential method for predicting pCR, but published false-negative rates remain variable and highly dependent on patient selection, imaging-pathology concordance and biopsy technique [43].

Several prospective studies are exploring whether surgery can be omitted safely in patients predicted to have achieved pCR after NST [44,45]. At present, however, this strategy remains investigational and should be confined to clinical trials or carefully governed prospective protocols. International collaboration, rigorous imaging–pathology correlation and standardised biopsy methods will be essential if this approach is to move towards routine practice.

## 6. Discussion

Over the past 130 years, breast cancer surgery has evolved toward progressively less extensive interventions, with the aim of achieving optimal oncological outcomes while preserving quality of life. This shift does not reflect simple surgical conservatism, but a robust evidence base derived from randomised trials, large prospective studies, guideline updates, and the systematic de-implementation of low-value surgical practices [4].

The contemporary landscape is characterised by the convergence of advances across multiple domains, including systemic therapy, imaging, pathology, radiotherapy, and surgical technique. Increasing rates of pathological complete response (pCR) following modern neoadjuvant regimens are enabling the de-escalation of both breast and axillary surgery in selected patient subgroups. At the same time, oncoplastic approaches permit wider resections while maintaining favourable aesthetic outcomes, and minimally invasive or robotic techniques aim to further improve postoperative recovery and patient experience.

Despite these advances, several challenges remain. Robotic mastectomy continues to be limited by cost, technical demands, and the absence of long-term oncological outcomes. The omission of surgery in exceptional responders represents an appealing but still investigational strategy, contingent on the development of highly accurate, non-invasive methods to confirm pCR. Similarly, the role of intraoperative radiotherapy (IORT) remains controversial, given concerns regarding local recurrence and cautious recommendations from current guidelines [46]. Artificial intelligence-based tools, although promising, are hindered by issues related to dataset bias, external validity, reproducibility, and integration into clinical workflows [47,48]. Antibody–drug conjugates are reshaping breast cancer systemic therapy and may indirectly affect surgical decisions as they are used earlier, but their role in surgical de-escalation is not yet established [49].

Future progress will likely depend on the integration of circulating biomarkers, advanced imaging modalities, image-guided tumour-bed assessment, next-generation systemic therapies, and increasingly personalised locoregional strategies. In this context, the key question for the coming decade will not be what surgery can technically be performed, but rather what surgery is truly necessary for an individual patient, taking into account tumour biology, treatment response, and patient preferences.

As a narrative review, this article does not aim to provide exhaustive systematic coverage. Instead, it offers a clinically oriented synthesis of the field, highlighting landmark studies, current guidelines, and emerging—yet still investigational—directions [50]. This review has several limitations. First, as a narrative review, it is subject to selection bias, as the included studies were chosen based on relevance rather than a predefined systematic methodology. Second, although priority was given to high-quality evidence, the synthesis remains interpretative and may reflect author perspective. Third, some of the emerging strategies discussed—such as omission of surgery after neoadjuvant therapy, robotic surgery, and novel intraoperative technologies—are supported by early-phase or non-randomized data, limiting the strength of conclusions regarding their role in surgical de-escalation. Fourth, heterogeneity across studies in terms of patient selection, tumour biology, and treatment protocols limits direct comparability. Finally, rapid technological evolution in breast cancer management means that some areas of evidence remain immature and subject to change with ongoing trials.

## 7. Conclusions

Breast cancer surgery has moved from a paradigm of maximum tolerable treatment to one of minimum effective intervention, guided by an expanding evidence base and by continuing technological innovation. Contemporary care is characterised by breast conservation when feasible, oncoplastic reconstruction, individualised axillary management and response-adapted planning in the neoadjuvant era. Emerging approaches—including robotic surgery, highly selective omission strategies, AI-supported planning and increasingly effective systemic therapies—may further refine treatment, but their adoption must remain anchored to prospective evidence and patient-centred outcomes.

## Data Availability

No new data were acquired during the preparation of the paper.
